# The Parkinson's Disease Mendelian Randomization Research Portal

**DOI:** 10.1002/mds.27873

**Published:** 2019-10-28

**Authors:** Alastair J. Noyce, Sara Bandres‐Ciga, Jonggeol Kim, Karl Heilbron, Demis Kia, Gibran Hemani, Angli Xue, Debbie A. Lawlor, George Davey Smith, Raquel Duran, Ziv Gan‐Or, Cornelis Blauwendraat, J. Raphael Gibbs, 23andMe Research Team, International Parkinson's Disease Genomics Consortium (IPDGC), David A. Hinds, Jian Yang, Peter Visscher, Jack Cuzick, Huw Morris, John Hardy, Nicholas W. Wood, Mike A. Nalls, Andrew B. Singleton

**Affiliations:** ^1^ Preventive Neurology Unit, Wolfson Institute of Preventive Medicine Queen Mary University of London London United Kingdom; ^2^ Department of Clinical and Movement Neurosciences University College London, Institute of Neurology London United Kingdom; ^3^ Molecular Genetics Section, Laboratory of Neurogenetics, National Institute on Aging National Institutes of Health Bethesda Maryland USA; ^4^ Instituto de Investigación Biosanitaria de Granada (ibs.GRANADA) Granada Spain; ^5^ 23andMe, Inc., Mountain View California USA; ^6^ MRC Integrative Epidemiology Unit University of Bristol Bristol United Kingdom; ^7^ Institute for Molecular Bioscience The University of Queensland Brisbane Australia; ^8^ Queensland Brain Institute The University of Queensland Brisbane Queensland Australia; ^9^ Population Health Science, Bristol Medical School University of Bristol Bristol United Kingdom; ^10^ Centro de Investigacion Biomedica and Departamento de Fisiologia, Facultad de Medicina Universidad de Granada Granada Spain; ^11^ Department of Neurology & Neurosurgery McGill University Montreal, Quebec Canada; ^12^ Montreal Neurological Institute McGill University Montreal, Quebec Canada; ^13^ Department of Human Genetics McGill University Montreal, Quebec Canada; ^14^ Institute for Advanced Research Wenzhou Medical University Wenzhou, Zhejiang China; ^15^ Data Tecnica International Glen Echo, Maryland USA

**Keywords:** Mendelian randomization, Parkinson's disease, public resource, risk factor

## Abstract

**Background:**

Mendelian randomization is a method for exploring observational associations to find evidence of causality.

**Objective:**

To apply Mendelian randomization between risk factors/phenotypic traits (exposures) and PD in a large, unbiased manner, and to create a public resource for research.

**Methods:**

We used two‐sample Mendelian randomization in which the summary statistics relating to single‐nucleotide polymorphisms from 5,839 genome‐wide association studies of exposures were used to assess causal relationships with PD. We selected the highest‐quality exposure genome‐wide association studies for this report (n = 401). For the disease outcome, summary statistics from the largest published PD genome‐wide association studies were used. For each exposure, the causal effect on PD was assessed using the inverse variance weighted method, followed by a range of sensitivity analyses. We used a false discovery rate of 5% from the inverse variance weighted analysis to prioritize exposures of interest.

**Results:**

We observed evidence for causal associations between 12 exposures and risk of PD. Of these, nine were effects related to increasing adiposity and decreasing risk of PD. The remaining top three exposures that affected PD risk were tea drinking, time spent watching television, and forced vital capacity, but these may have been biased and were less convincing. Other exposures at nominal statistical significance included inverse effects of smoking and alcohol.

**Conclusions:**

We present a new platform which offers Mendelian randomization analyses for a total of 5,839 genome‐wide association studies versus the largest PD genome‐wide association studies available (https://pdgenetics.shinyapps.io/MRportal/). Alongside, we report further evidence to support a causal role for adiposity on lowering the risk of PD. © 2019 The Authors. *Movement Disorders* published by Wiley Periodicals, Inc. on behalf of International Parkinson and Movement Disorder Society.

Although monogenic forms of Parkinson's disease (PD) are responsible for approximately 5% of cases, the vast majority of disease is considered to be sporadic and attributable to a range of genetic and nongenetic risk factors.^1^ Total heritability estimates for PD are 22% to 27%, with approximately one‐third of this explained by genome‐wide association studies (GWAS) and a substantial proportion of genetic risk still to be discovered.^2^, ^3^, ^4^, ^5^ The remainder of PD risk comes from environmental factors,^4^, ^5^ aging,^6^, ^7^ and stochastic events.^8^

Mendelian randomization (MR) is an epidemiological method that can be used to provide support for causality between a modifiable exposure/risk factor/phenotypic trait (henceforth collectively termed *exposure*) and a disease outcome.^9^ Put simply, genetic variants (usually single‐nucleotide polymorphisms [SNPs]) that explain variation in an exposure can be used as proxies to determine how a change in that exposure might influence a disease outcome. A ratio of the genetically estimated change in the exposure and the genetically estimated change in the outcome using the same individual SNP is calculated and then the ratios are pooled across all SNPs that are independently associated with the exposure of interest. The pooled ratio is an estimate of change in the outcome for a given change in the exposure, as long as certain instrumental variable assumptions are upheld (see Supporting Information).

A common approach to MR involves the use of summary statistics from published GWASes of exposures and the summary statistics of a GWAS of an outcome; an approach known as two‐sample MR. Recently, the summary statistics from GWAS for a large range of exposures have been curated in *MR Base* (http://www.mrbase.org), which enables targeted (hypothesis‐driven) exploration of causal associations or hypothesis‐generating approaches to MR.^10^ We have undertaken two‐sample MR for a wide range of exposures and PD. The principal goal was to provide a new resource for the research community to add causal insights to associations arising from traditional epidemiological approaches and to support the pursuit of new interventions to reduce the risk of PD.

## Materials and Methods

### Exposure Data

*MR Base* is an online resource which, at the time of the analysis, contained summary results from 7,956 GWASes across multiple exposures, encompassing a wide range of physiological characteristics and disease phenotypes. *MR Base* was accessed on January 14, 2019. Each exposure was tested separately to determine whether it altered risk of PD. All analyses were performed using the R package, *TwoSampleMR* (version 3.2.2; https://github.com/MRCIEU/TwoSampleMR). The instrumental variables used for each binary exposure consisted of the per‐allele log‐odds ratio (or the beta estimate for continuous exposures) and standard errors for all independent GWAS significant SNPs.

We used the following stringent criteria for any exposure GWAS to be included in our analysis: (1) the GWAS had to report SNPs with *P* values <5.0 × 10^–8^ for their association with a given exposure; (2) these SNPs or their proxies (linkage disequilibrium R^2^ value > = 0.8) had to be present in both the exposure and outcome (PD) data sets; and (3) these SNPs were independent signals that were generated through a process called “clumping.” In order to “clump,” index SNPs were identified by ranking exposure associations from the smallest to largest *P* value (but still with a cut‐off value of *P =* 5 × 10^–8^). Clumped SNPs were those in linkage disequilibrium (LD) with index SNPs (R^2^ threshold of 0.001) or within 10,000 kb physical distance. Hence, each index SNP represented a number of clumped SNPs that were all associated with or near to the index SNP, and the index SNPs were all independent of one another (according to the stringent parameters defined here). A total of 5,839 exposure GWASes surpassed these criteria and were tested against the outcome. Then, we further expanded our filtering approach as follows: (4) in order to use MR sensitivity analyses designed to identify pleiotropy and its effects, each GWAS had to include a minimum of 10 associated SNPs; (5) the number of cases was >250 for GWASes of a binary exposure or > 250 individuals for GWASes of a continuous exposure; and (6) both the exposure and the outcome data were drawn from European populations. A total of 401 exposures met our filtering criteria (7% of 5,839), consisting of 175 published GWASes and 226 unpublished GWASes from the UK Biobank (UKB; http://www.ukbiobank.ac.uk/). For UKB GWASes, some of the exposures are reported in an ordinal fashion, but treated as continuous when calculating betas for the effect allele at each SNP. This means that some of the effect estimates that arise are difficult to interpret quantitatively, both in the GWAS and in the subsequent MR analysis.

### Outcome Data

Summary statistics from the largest, published PD GWAS meta‐analysis involving 26,035 PD cases and 403,190 controls of European ancestry were used as the outcome data for the primary analysis. Recruitment and genotyping quality‐control procedures were described in the original report.^11^

A newer PD GWAS included a total of 37,688 cases, 1,417,791 controls, and 18,618 “proxy cases” from the UKB (individuals that reported having a parent with PD).^2^ However, there was substantial overlap in control subjects between each of the UKB exposures and the Nalls and colleagues 2019 meta‐analysis, which can, in turn, lead to bias in causal effect estimates. For this reason, we repeated the analyses using only 5,851 clinically diagnosed PD cases and 5,866 matched controls as the outcome, after excluding UKB samples and self‐reported PD cases and controls. Finally, we used an earlier PD GWAS as the outcome that included 13,708 cases and 95,282 controls.^12^

### MR Analyses

Harmonization was undertaken to rule out strand mismatches and to ensure alignment of SNP effect sizes. Wald ratios were calculated for each clumped SNP in the exposure GWAS by dividing the per‐allele log‐odds ratio (or beta) of that variant in the PD GWAS data by the log‐odds ratio (or beta) of the same variant in the exposure data.

First, the inverse‐variance weighted (IVW) method was implemented to examine the relationship between the individual exposures and PD. In this method, the Wald ratio for each SNP was weighted according to its inverse variance and the effect estimates were meta‐analyzed using random effects. This approach is equivalent to plotting SNP‐exposure/SNP‐outcome associations on scatter plot and fitting a regression line (IVW regression), which is constrained to pass through the origin. The slope of the linear regression represents the pooled‐effect estimate of the individual SNP Wald ratios.^13^ For the purpose of demonstrating the use of this new platform, we used a false discovery rate (FDR)‐adjusted *P* value of <0.05 to define exposures of interest as showing potential evidence of a causal effect. The IVW estimate is valid when the three core assumptions that underpin MR are upheld (see Supporting Information). However, simulation studies show that up to 90% of MR analysis may be affected by pleiotropy, which, in turn, may bias the IVW estimate.^14^ Effects of pleiotropy for each analysis were studied by first looking for evidence of heterogeneity in the SNP Wald ratios and then undertaking a range of sensitivity analyses, each with different underlying assumptions.

Heterogeneity in the IVW estimates was tested using Cochran's Q test, quantified using the I^2^ statistic, and displayed in forest plots. Heterogeneity in the IVW estimate may indicate that alternative pathways exist from some of the SNPs to the outcome (known as horizontal pleiotropy), which can violate the third MR assumption,^15^ but as long as overall heterogeneity is balanced it does not necessarily bias the pooled IVW estimate.^16^ After calculation of the IVW estimates, three sensitivity analyses were applied to evaluate the core assumptions of MR. These rely on instruments containing multiple SNPs (in this case a minimum of 10 SNPs per exposure).^17^

MR‐Egger was used, in which the regression line fitted to the data is not constrained to pass through the origin and a nonzero intercept indicates whether there is a net horizontal pleiotropic effect which may bias the IVW estimate.^18^ The weighted median (WM) MR method gives consistent effect estimates under the assumption that no more than 50% of the weight of the MR effect estimate comes from invalid (e.g., pleiotropic) SNPs, where weight is determined by the strength of their association with the exposure.^19^ Finally, IVW radial analysis was performed as a complementary method to account for SNPs acting as heterogeneous outliers and to determine the effect of resulting bias on the IVW estimate.^16^

### Exploring Directionality, Single SNP Effects, and Reverse Causality

For effect estimate directionality, odds ratios were scaled on a standard deviation increase in genetic risk for the exposure from that population mean. We evaluated the possibility that the overall estimate was driven by a single SNP using leave‐one‐out (LOO) analyses for each of the exposures associated with PD. Finally, we tested for reverse causation by using SNPs tagging the independent loci described in the latest PD GWAS as exposure instrumental variables and exposure GWASes as the outcomes. Note that this analysis measures the causal effect of genetic liability toward PD on each of the exposures included in the main analysis, which is independent of PD actually occurring (in a case‐control setting such as this).

## Results

**Figure 1:**
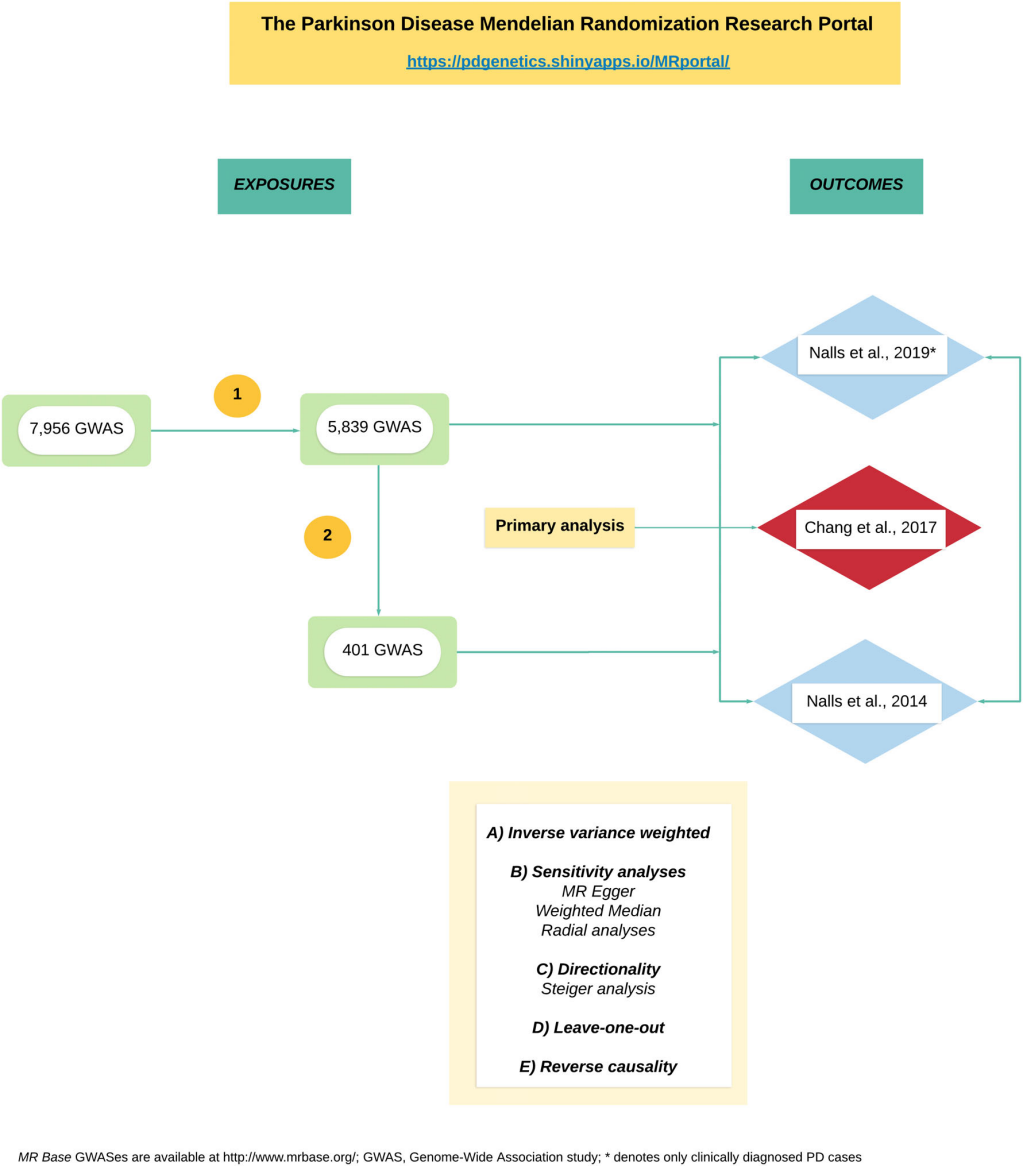
Flowchart of analysis. The PD MR Research Portal is an interactive tool where the user can explore causal associations across multiple exposures. (1) The inclusion criteria used include (i) GWASes with associated SNPs with *P* values <5.0 × 10^–8^; (ii) SNPs present in both the exposure and outcomes (Chang and colleagues 2017 as the primary analysis, Nalls and colleagues 2019 with only clinically diagnosed cases, and Nalls and colleagues 2014) data sets or when not present their LD proxies (R2 value: > = 0.8); and (iii) independent SNPs (R2 < 0.001 with any other associated SNP within 10 Mb), considered as the most stringent clumping threshold used when performing MR analyses. A total of 5,839 GWASes surpassed this criteria and were tested against the three outcomes. (2) Then, we further expanded our filtering approach as follows: (iv) each GWAS had to include a minimum of 10 associated SNPs in order to use MR sensitivity analyses designed to identify pleiotropy; (v) the number of cases had to be >250 for each GWAS of a given binary exposure or > 250 individuals for each GWAS of a given continuous exposure; and (vi) the exposure and the outcome data sets were drawn from European populations. A total of 401 exposures surpassed our filtering approach consisting of 175 published GWASes and 226 unpublished GWASes from the UKB (http://www.ukbiobank.ac.uk). [Color figure can be viewed at http://wileyonlinelibrary.com]

The PD MR Research Portal is hosted at https://pdgenetics.shinyapps.io/MRportal/. Here, we explore some of the top results to assist users in understanding how to interpret these data (see Fig. 1 for flowchart of analysis).

Of the 401 exposures that survived the filtering process, we found 12 exposures with potentially causal effects on PD (i.e., IWV FDR‐adjusted *P* values <0.05; see Table 1 and Supporting Information Fig. S1 for forest plots displaying individual SNP‐level estimates and pooled estimates). Of these exposures, nine were measures of adiposity that implied an inverse causal effect of increase adiposity and a lowering of PD risk. Three additional exposures met the FDR‐adjusted *P*‐value threshold for a possible causal effect: time spent watching television (inverse), tea drinking (positive), and forced vital capacity (FVC; positive). In each case, to explore the possibility that results were biased because of the violation of core MR assumptions, we looked for heterogeneity in the individual Wald ratios and performed the three additional MR sensitivity analyses (see Table 2 for heterogeneity analyses and Table 1 for sensitivity analyses).

**Table 1:** Mendelian randomization analyses of exposures with IVW FDR < 0.05 vs Chang et al., 2017

		IVW				MR Egger			Weighted Median			Radial IVW		
Exposure	no. of SNPs	beta	se	p‐value	FDR	beta	se	p‐value	beta	se	p‐value	beta	se	p‐value
Arm fat percentage (right) | | id:UKB‐a:282	214	−0.431	0.098	1.10E‐05	0.005	−0.657	0.324	0.044	−0.372	0.143	0.009	−0.454	0.079	3.35E‐08
Arm fat percentage (left) | | id:UKB‐a:286	233	−0.417	0.103	4.94E‐05	0.010	−0.790	0.333	0.018	−0.201	0.137	0.143	−0.434	0.077	5.41E‐08
Arm fat mass (right) | | id:UKB‐a:283	248	−0.276	0.072	1.32E‐04	0.011	−0.305	0.218	0.164	−0.299	0.100	0.003	−0.271	0.056	2.44E‐06
Leg fat percentage (right) | | id:UKB‐a:274	228	−0.482	0.126	1.33E‐04	0.011	−0.944	0.479	0.050	−0.436	0.158	0.006	−0.465	0.096	2.54E‐06
Whole body fat mass | | id:UKB‐a:265	258	−0.269	0.070	1.35E‐04	0.011	−0.362	0.220	0.102	−0.241	0.102	0.018	−0.281	0.056	7.95E‐07
Arm fat mass (left) | | id:UKB‐a:287	248	−0.279	0.074	1.58E‐04	0.011	−0.492	0.222	0.027	−0.307	0.105	0.004	−0.288	0.056	6.07E‐07
Trunk fat mass | | id:UKB‐a:291	257	−0.259	0.070	2.10E‐04	0.012	−0.438	0.227	0.055	−0.256	0.094	0.007	−0.255	0.054	4.37E‐06
Time spent watching television (TV) | | id:UKB‐b:5192	104	−0.775	0.211	2.40E‐04	0.013	−0.166	1.016	0.870	−0.586	0.234	0.012	−0.772	0.156	2.81E‐06
Trunk fat percentage | | id:UKB‐a:290	216	−0.307	0.085	3.20E‐04	0.015	−0.554	0.307	0.073	−0.217	0.111	0.050	−0.325	0.066	1.49E‐06
Forced vital capacity (FVC) Best measure | | id:UKB‐a:232	179	0.441	0.128	5.80E‐04	0.024	0.940	0.381	0.015	0.241	0.123	0.050	0.455	0.075	6.27E‐09
Tea intake | | id:UKB‐b:6066	35	0.554	0.163	6.58E‐04	0.025	0.710	0.334	0.041	0.543	0.223	0.015	0.570	0.143	2.97E‐04
Impedance of leg (right) | | id:UKB‐a:270	294	0.220	0.066	9.17E‐04	0.032	0.212	0.189	0.263	0.315	0.091	0.001	0.233	0.054	2.14E‐05

id, specific code attributed to each trait by MR Base; se, standard error; MR, Mendelian randomization; IVW, Inverse Variance Weighted; FDR, false discovery rate adjusted p value.

**Table 2:** Heterogeneity, horizontal pleiotropy and directionality analyses for exposures with FDR < 0.05 vs Chang et al. 2017

	Horizontal pleiotropy			Heterogeneity						Directionality
Exposure	Egger intercept	se	p ‐value	MR Egger Q	MR Egger Q df	MR Egger p‐value	IVW Q	IVW Q df	IVW p‐value	
Arm fat percentage (right) | | id:UKB‐a:282	3.684E‐03	0.005	0.466	314.024	212	6.521E‐06	314.816	213	7.006E‐06	−
Arm fat percentage (left) | | id:UKB‐a:286	5.959E‐03	0.005	0.239	399.388	231	3.926E‐11	401.794	232	3.090E‐11	−
Arm fat mass (right) | | id:UKB‐a:283	6.229E‐04	0.004	0.887	394.669	246	5.351E‐09	394.701	247	6.786E‐09	−
Leg fat percentage (right) | | id:UKB‐a:274	5.925E‐03	0.006	0.319	374.029	226	2.108E‐09	375.682	227	1.951E‐09	−
Whole body fat mass | | id:UKB‐a:265	1.975E‐03	0.004	0.656	399.929	256	2.122E‐08	400.239	257	2.523E‐08	−
Arm fat mass (left) | | id:UKB‐a:287	4.548E‐03	0.004	0.309	408.501	246	3.374E‐10	410.229	247	3.076E‐10	−
Trunk fat mass | | id:UKB‐a:291	3.900E‐03	0.005	0.408	412.287	256	2.001E‐09	411.179	255	1.942E‐09	−
Time spent watching television (TV) | | id:UKB‐b:5192	−7.167E‐03	0.012	0.542	186.037	102	7.452E‐07	186.721	103	8.687E‐07	−
Trunk fat percentage | | id:UKB‐a:290	4.814E‐03	0.006	0.405	352.411	214	7.667E‐09	353.559	215	7.863E‐09	−
Forced vital capacity (FVC) Best measure | | id:UKB‐a:232	−9.804E‐03	0.007	0.166	497.966	177	2.655E‐32	503.407	178	7.621E‐33	+
Tea intake | | id:UKB‐b:6066	−3.608E‐03	0.007	0.596	40.971	33	1.605E‐01	41.327	34	1.811E‐01	+
Impedance of leg (right) | | id:UKB‐a:270	1.645E‐04	0.004	0.965	427.081	292	4.081E‐07	427.084	293	4.972E‐07	+

id, specific code attributed to each trait by MR Base; se, standard error; IVW, Inverse variance weighted; MR, Mendelian randomization; Q, Cochran's Q test estimate; df, Cochran's Q test degrees of freedom.

The eight direct adiposity GWASes all contained >200 SNPs and were highly correlated with one another. The strongest causal effects were observed for arm fat percentage, which was measured using tissue impedance (https://biobank.ctsu.ox.ac.uk/crystal/field.cgi?id=23119). A unit increase in arm fat percentage (right and left) yielded a pooled odds ratio (OR) of 0.65 (95% confidence interval [CI]: 0.54–0.79; *FDR adjusted P* = 0.005) and a pooled OR of 0.66 (95% CI: 0.54–0.79; *FDR adjusted P* = 0.01), respectively. Although the individual Wald ratios showed significant heterogeneity (arm fat percentage [right] Q = 314.8; *P* = 7.01 × 10^–6^; arm fat percentage [left] Q = 399.38; *P* = 3.93 × 10^–11^), the sensitivity analyses did not suggest significant bias in the causal effect estimate from the IVW analysis (see Table 2 for heterogeneity analyses and Table 1 for sensitivity analyses). In general, all effect estimates for the adiposity exposures supported a protective effect of increased adiposity on risk of PD (ORs ranged between 0.62 and 0.77; see Table 1).

Another adiposity exposure called “impedance of right leg” surpassed the IVW FDR‐adjusted *P*‐value threshold. As described above, impedance is the method by which percentage fat is calculated, with higher impedance indicative of higher fat percentage (https://biobank.ctsu.ox.ac.uk/crystal/field.cgi?id=23107). Given the results for adiposity, we expected that a unit change in impedance would result in a lowering of PD risk. In contrast to the results for adiposity, the impedance exposure gave rise to an IVW OR of 1.25 (95% CI: 1.10–1.42; *FDR adjusted P* = 0.032). There was significant heterogeneity in the individual Wald ratios (Q = 428.4; *P* = 7.25 × 10^–7^), but the sensitivity analyses did not suggest bias in the IVW effect estimate (see Table 2 for heterogeneity analyses and Table 1 for sensitivity analyses). We sought to explain why the direction of effect for impedance was different to that for percentage fat, when it was expected that it should be the same. Genetic correlations between impedance and percentage fat were run (see Supporting Information Table S1), and in all cases the genetic association between the two exposures was negative.

The “tea drinking” exposure was captured by an instrument containing 35 SNPs. The IVW OR was 1.74 (95% CI: 1.26–2.39; *FDR adjusted P* = 0.025), and there was no significant heterogeneity in the individual Wald ratios (Q = 41.3; *P* = 0.181). The sensitivity analyses did not imply bias in the IVW estimate. Of note, coffee intake was not causally associated with increased risk of PD (OR, 1.37; *P* = 0.197), but similar to the tea drinking exposure, the direction of effect was also not consistent with the recognized negative observational effect.

“Time spent watching television” was inversely linked to PD risk in the IVW analysis (OR, 0.46; *FDR adjusted P* = 0.013), suggesting that more time spent watching television caused a lower risk of PD. However, the MR Egger sensitivity analysis gave a very different pooled effect estimate (OR, 0.85; *P* = 0.870), suggesting that the IVW result may have been biased by directional pleiotropy. The intercept term can be used to test for net directional pleiotropy (here it was –0.007; *P* = 0.542), but this test is generally underpowered. The difference in the slope from the IVW analysis and the MR Egger analysis is shown graphically in Supporting Information Figure S2, and suggests that, in the presence of directional pleiotropy, the IVW effect for time spent watching television may be overestimated. From the other scatter and forest plots, it is clear that for the adiposity exposures, that the slope (or magnitude of effect) the MR Egger regression is greater than the IVW slope (effect), which suggests that in the presence of directional pleiotropy, the IVW may be underestimated.

“Forced vital capacity” showed a positive causal effect on PD risk that was similarly observed in the sensitivity analyses (see Table 1). However, the effects appeared to be largely driven by two SNPs known to be pleiotropic for PD (see below).

The LOO analysis showed that none of the results described for the 12 exposures were explained by a single SNP in each of the instruments (Supporting Information Table S2). The most precisely estimated Wald ratio for most of the adiposity exposures (seven of eight) came from a single SNP in the *FTO* gene (rs11642015), but dropping this SNP from the analyses did not affect the overall results. Similarly, the most precisely estimated Wald ratio in the impedance instrument was for a different SNP in the *FTO* gene (rs62048402), but again dropping this SNP did not affect the overall results. Importantly, and in support of the observations relating to negative genetic correlation between percentage fat and impedance described above, the direction of the Wald ratio for the *FTO* SNP in adiposity exposures was negative and for impedance was positive.

For tea drinking, the two Wald ratios with the greatest influence on the pooled effect estimates came from SNPs rs4410790 and rs2472297 (located in the *AHR* and *CYP1A2* gene loci respectively), which are known to be strongly associated with caffeine consumption.^20^ Leaving either SNP out from the analysis did not change the overall result, but the pooled effect estimate weakened when rs4410790 (*AHR*) was removed (the OR 1.74 changed to 1.61). In the forced vital capacity analysis, SNPs rs1991556 and rs13146142 were most precisely estimated and appeared to influence the magnitude of the causal effect. Closer examination revealed that rs1991556 is in the *MAPT* locus and rs13146142 is in the *LCORL* locus, and both are known to be associated with PD, likely biasing the causal effect of FVC on PD.^2^, ^12^

The reverse causation analyses revealed no clear evidence that a liability toward PD was causally linked with any of the 12 exposures, but this analysis was restricted to only 18 of the 43 PD GWAS significant hits and may have been underpowered (Supporting Information Table S3).

Of interest, the next seven exposures with the strongest associations in the IVW analysis, but not surpassing the FDR‐adjusted *P*‐value threshold, were four adiposity traits (all showing a negative causal effect), current tobacco smoking (negative causal effect), increased alcohol intake (negative causal effect), and increased education (having a college or university degree; positive causal effect). The FDR‐adjusted *P* values for each of these were < 0.1 and unadjusted *P* values were all <0.004.

Finally, we used clinically diagnosed cases (and controls) from the 2019 PD GWAS dataset as the outcome (Supporting Information Table S4). Given the smaller sample size, none of the MR analyses surpassed an FDR‐adjusted *P* value, but the top hit was a marker of adiposity (hip circumference). When an even earlier iteration of the PD GWAS was used (~13.5k cases and ~95k controls),^12^ there were 11 exposures that surpassed the FDR‐adjusted *P*‐value threshold and all 11 were related to adiposity (Supporting Information Table S5).

## Discussion

We envisage the PD MR Research Portal being a valuable and evolving resource for the global research community, which will be updated as new data emerge. It should be used to provide evidence to support, and, over time, evidence against, causality when undertaking observational studies or pursuing interventions aimed at reducing the risk of PD. Clearly, there are too many associations presented in the portal to explore each one in detail. So, for the purpose of demonstrating the tool, we undertook a data‐driven approach to identify those exposures with the strongest causal signals.

We have previously reported an inverse causal association between body mass index (BMI) and risk of PD—a genetically estimated 5 kg/m^2^ higher BMI was associated with a reduced risk of PD (IVW OR = 0.82).^21^ Here, we found further evidence to support an inverse relationship between increased adiposity and PD, given that eight of the top 12 exposures were measures of adiposity. These exposures were objectively and quantitatively ascertained, and each GWAS identified >200 SNPs that were associated with increased adiposity. The results of the IVW analyses and all sensitivity analyses were broadly consistent. Impedance (the ninth adiposity exposure in the analysis) gave rise to a causal effect opposite to that of increasing the other adiposity exposures, which was initially unexpected. The difference in the direction of effect may be because the equation that links percentage fat and impedance requires adjustment for height and weight, which would not have been done routinely in the impedance GWAS.^22^

Of note, although BMI was not one of the top 12 exposures, the causal effect of BMI on PD was also negative in this analysis (OR, 0.81; *P* = 0.037), using the same BMI instrument that we have previously published on.^21^, ^23^ In that earlier analysis, we included simulations that indicated that the protective effect was unlikely to be explained by survival bias (i.e., people with higher BMI dying of other diseases before they would usually be diagnosed with PD). The relationship between BMI and adiposity with risk of PD clearly warrants further study, especially given uncertainty in the role of BMI arising from traditional observational studies.^21^, ^24^

We found no evidence to support a protective effect of coffee and tea drinking, and instead unexpectedly found some evidence that tea drinking may increase the risk of PD. Observational study associations between caffeine‐containing drinks (both coffee and tea) have consistently suggested a negative association with PD, and caffeine has been explored as a potentially therapeutic option for PD.^4^, ^25^, ^26^ Our results should not be considered in isolation given a large body of observational evidence, which has been amassed from well‐conducted prospective studies using detailed dietary questionnaires and long durations of follow‐up. Clearly, using a genetic instrument to capture a behavior reported by a question in the UKB survey may be flawed compared to the traditional approaches outlined above. However, the current findings perhaps provide some indirect support for the notion that negative associations between caffeine consumption and PD may be driven by reverse causation rather than a true protective effect, and this warrants further study.

In contrast to tea drinking, there was some evidence to support a true protective effect of smoking, which fell below the threshold to be discussed as a top result. The remaining two exposures (time spent watching television and forced vital capacity) that appeared to be causally linked to PD should be regarded with caution. Television watching failed in one of the sensitivity analyses and the effect of forced vital capacity appeared to be driven, in part, by two recognized PD SNPs, rendering these two results potentially invalid.

Alongside a standard method used to pool ratio estimates from individual SNPs (the IVW method), we also demonstrated the use of three sensitivity analyses that provide more valid estimates when core instrumental variable assumptions have been violated (namely the MR‐Egger, WM, and radial methods).^16^, ^19^ These methods will allow researchers to further appraise the validity of the instruments presented and whether IVW estimates may be biased. In the current version of the portal, we have provided the opportunity of undertaking analyses with three different PD outcome data sets. There was little qualitative difference in the “top MR hits” across the three iterations of the PD outcome data, particularly when comparing the larger data sets. The portal also contains a huge number of exposures that have been curated by *MR Base*. However, as highlighted in this report and in a warning message in the portal, we advise caution with the interpretation of results for exposures that do not surpass considered filtering criteria (for our criteria, this was 5,438 of 5,839 or 93% of exposures). These included GWASes for which the case numbers were small and the number of variants in the exposure instruments was too few to permit the use of the sensitivity analyses.

### Limitations

The general limitations of MR have been covered extensively elsewhere,^9^ but include issues of statistical power (large sample sizes are required for these analyses), selection bias (which may arise through population stratification and is mitigated here by using only European ancestry GWASes), survival bias (particularly with age‐related outcomes such as PD, which we have discussed above), overlapping control groups (which may bias effect estimates, but have been avoided here), and violation in underlying MR assumptions (which are tested by a range of sensitivity analyses). In most instances, the variation in a given exposure that is explained by genome‐wide significant GWAS signals is small (i.e., 0.5–8%), meaning that sample sizes often need to be extremely large to detect causal effects using this design. The resulting *P* values that arise from MR hypothesis tests are often not small and, in the various sensitivity analyses, often border on or exceed nominal statistical significance. Given the approach, traditional methods of adjusting for multiple comparisons (i.e., Bonferroni or FDR correction) render many potentially causal associations obsolete and limit the conclusions that one can draw. In general, we recommend using this tool to confirm or add to evidence to associations identified in observational studies or toward a particular intervention. It is generally recommended that emphasis should be placed on the confidence intervals and consistency of point estimates across the IVW estimate and the sensitivity analyses, rather than just the *P* values *per se*. In addition, we recommend the calculation of F‐statistics for determining instrument strength and post‐hoc power calculations, particularly in the instance of null results.^2^, ^27^ This is particularly important when making claims of *no evidence* to support a causal association (such as in some of the reverse causation analyses presented in this article). These additions, and others, are planned for future versions of the portal.

For all analyses presented here, the outcome is “risk of PD” because the outcome data come from a GWAS of PD cases versus controls. In order to make causal inferences about the effect of various exposures on disease progression, one would need to have an outcome GWAS of PD progression. As mentioned in the Materials and Methods, an important source of bias in MR studies can occur when there is overlap in the control groups in the exposure and outcome data. For this reason, all UKB data were removed from the outcome PD GWAS summary statistics.

In summary, we present a new portal for use by the research community that will help assess causality where observational associations exist and prioritize (or deprioritize) interventions aimed at reducing risk of PD.

## Author Roles

(1) Research Project: A. Conception, B. Organization, C. Execution; (2) Data Generation: A. Experimental; (3) Statistical Analysis: A. Design, B. Execution, C. Review and Critique; (4) Manuscript Preparation: A. Writing of the First Draft, B. Review and Critique.

A.J.N.: 1A, 1B, 1C, 3A, 3B, 4A, 4B

S.B.‐C.: 1A, 1B, 1C, 3A, 3B, 4A, 4B

J.K.: 1A, 1B, 1C, 3A, 3B, 4B

K.H.: 3C,4B

D.K.: 3C,4B

G.H.: 3C,4B

A.X.: 3C,4B

D.A.L.: 3C,4B

G.D.S.: 3C,4B

R.D.: 3C,4B

Z.G‐O.: 3C,4B

C.B.: 3C,4B

J.R.G.: 3C,4B

23andMe Research Team: 2A

IPDGC: 2A

D.A.H.: 3C,4B

J.Y.: 3C,4B

P.V.: 3C,4B

J.C.: 3C,4B

H.M.: 3C,4B

J.H.: 3C,4B

N.W.W.: 3C,4B

M.A.N.: 2A,3C,4B

A.B.S.: 3C,4B

## Financial Disclosures

AJN reports grants from Parkinson's UK, Barts Charity, Leonard Wolfson Experimental Neurology Centre, UCL Movement Disorders Centre and the Virginia Kieley Benefaction; honoraria or consultancy fees from Britannia, Global Kinetics Corporation, Profile Pharmaceuticals, Guide point, Biogen and Roche. KH and DAH are employees of 23andMe and hold stock or stock options in 23andMe. DAL reports grants from the Medical Research Council, numerous charitable funders,Medtronic and Roche. ZG‐O reports consultancy fees from Inceptions Sciences,Idorsia, Denali, Lysosomal Therapeutics inc. HM reports reports consultancy from Biogen, UCB, Abbvie, Denali, Biohaven; lecture fees/honoraria from Biogen, UCB,C4X Discovery, GE‐Healthcare, Welcome Trust, Movement Disorders Society; Research Grants from Parkinson's UK, Cure Parkinson's Trust, PSP Association, CBD Solutions, Drake Foundation, Medical Research Council. Dr Morris is a co‐applicanton a patent application related to C9ORF72 (PCT/GB2012/052140).

## Supporting information


**Figure S1.** Forest plots showing point estimates of the exposures of interest.
**Figure S2.** Funnel plots showing point estimates of the exposures of interest.
**Table S1.** Genetic correlations between fat related traits and impedance related traits.
**Table S2.** Leave‐one‐out analyses of exposures of interest.
**Table S3.** Reverse causality analyses.
**Table S4.** Mendelian Randomization analyses of exposures significantly linked to PD at IVW p‐value <0.05 vs Nalls et al., 2019 (only clinically diagnosed cases).
**Table S5.** Mendelian Randomization analyses of exposures significantly linked to PD at IVW p‐value <0.05 vs Nalls et al., 2014.
